# In Situ Lipid Interactions of an Anticancer Metal Complex

**DOI:** 10.1021/acs.inorgchem.6c00104

**Published:** 2026-03-13

**Authors:** Edward C. Lant, Archana C. Jadhav, Annabel Sumeray, Gustavo F. Trindade, Luca Craciunescu, Andrew W. Prentice, Juliusz A. Wolny, Jaspreet S. Grewal, Robert Dallmann, Guy J. Clarkson, Ann M. Dixon, Volker Schünemann, Ian S. Gilmore, Martin J. Paterson, Maria Harkiolaki, Peter J. Sadler

**Affiliations:** † Department of Chemistry, 2707 University of Warwick, Coventry CV4 7AL, United Kingdom; ‡ 120796 Diamond Light Source, Harwell Science and Innovation Campus, Harwell OX11 0DE, United Kingdom; § 9917 National Physical Laboratory (NPL), Hampton Road, Teddington, Middlesex TW11 0LW, United Kingdom; ∥ School of Engineering & Physical Sciences, 3120 Heriot-Watt University, Edinburgh EH14 4AS, United Kingdom; ⊥ Department of Physics, 26562 RPTU University Kaiserslautern-Landau, Erwin-Schrödinger-Straße 46, 67663 Kaiserslautern, Germany; # Division of Biomedical Sciences, University of Warwick, Coventry CV4 7AL, United Kingdom

## Abstract

An integrated multimodal imaging workflow of cryogenic super-resolution fluorescence microscopy and soft X-ray tomography, Orbitrap secondary ion mass spectrometry, and inductively coupled plasma-mass spectrometry has revealed the unexpected targeting of a half-sandwich cyclopentadienyl Rh­(III) phenylazopyridine anticancer complex to cellular lipid membranes and lipid droplets. The complex accumulates in plasma membranes with a surprisingly intense switch-on luminescence in living cancer cells, drives remodeling of lipid droplet architecture, and penetrates deeply into lipid-rich tissue environments. DFT modeling shows strong supramolecular interactions between the complex and glycer­ophos­phoryl­choline lipids.

## Graphical abstract

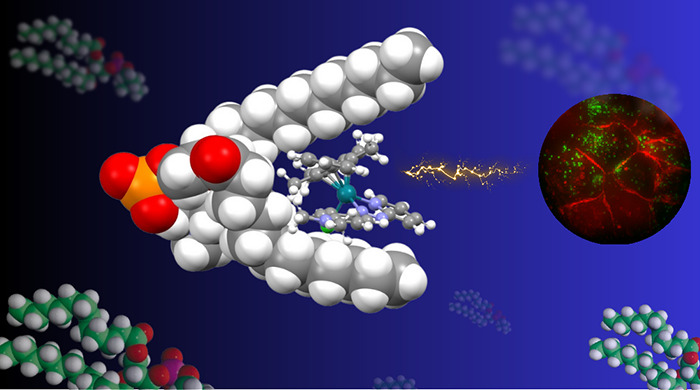

Metal-based therapeutic agents, in particular organometallic complexes, offer promising opportunities for the discovery of novel drugs.^1,2^ Progress is hampered by the need to identify their molecular targets and modes of action.^2–4^ Many metallodrugs are pro-drugs which undergo ligand exchange and redox reactions in complicated biological media.^5^ Hence mapping the chemical species which are their active pharmacophores is important. Recent advances in multimodal and integrative analytical approaches provide the means to interrogate these species and their interactions with unprecedented spatial, chemical, and mechanistic resolution.^6,7^ These include high resolution luminescence imaging, soft- and hard-X-ray imaging,^7^ secondary ion mass spectrometry,^8,9^ and inductively coupled plasma-mass spectrometry.^10^ Important is the emergence of methods to study intact cryo-fixed cells since chemical fixatives and other treatments can affect metal speciation.^7^ The combination of advances in metal speciation together with genomics and proteomics (“metallomics”) offers major advances in systems pharmacology for metallodrugs.^10–12^

Here we study a low-spin pseudo-octahedral 4d^6^ Rh­(III) complex coordinated to a π-bonded η^5^-cyclopentadienyl, a *N*,*N*-chelated phenylazopyridine and monodentate chlorido ligands, [(η^5^-Cp*)­Rh­(Me_2_ *N*-phenyl­azo­pyri­dine)­Cl]^+^, **1** (Figure 1a). The complex exhibits micromolar cytotoxic potency across multiple cancer cell lines, with low toxicity toward normal cells, and no cross-resistance with cisplatin.^13^ The azo-bond is locked into the *trans*-E configuration (Figure 1b), unlike the free ligand. The parent phenylazopyridine ligand is a nonemissive azo dye and a safe anti-infective drug (Phenazopyridine).^14^

**Figure 1:**
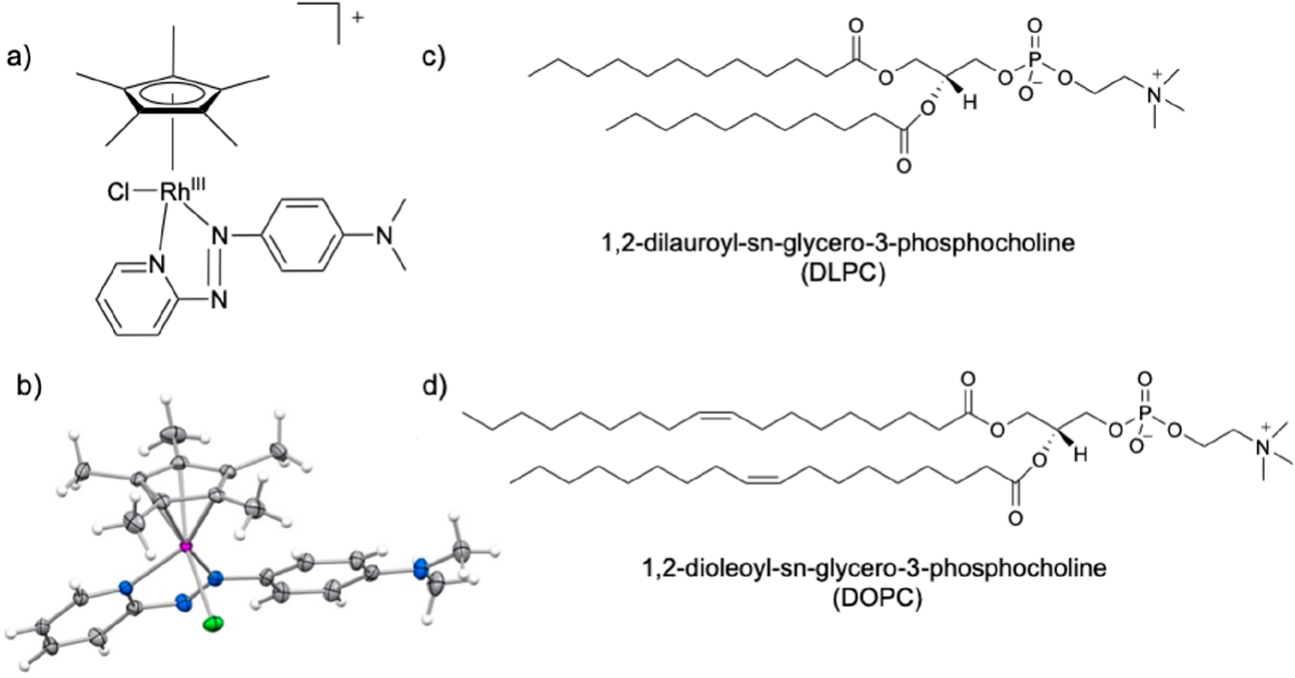
Complex **1** and phospholipids. (a) [η^5^-Cp*)­Rh­(4-Me_2_ *N*-phenyl­azpy)­Cl]^+^; (b) X-ray crystal structure of **1**.PF_6_, thermal ellipsoids drawn at 50% probability, counteranion omitted, showing the *trans* (E) N=N azo bond configuration. Rh, purple; N, blue; Cl, green. Phospholipids (c) DLPC and (d) DOPC.

To elucidate the intracellular chemistry of **1** and mode of action, we implemented a multimodal correlative imaging workflow combining super-resolution cryogenic structured illumination microscopy (cryoSIM), soft X-ray tomography (SXT) on vitrified cancer cells, inductively coupled plasma-mass spectrometry (ICPMS), and Orbitrap secondary ion mass spectrometry (OrbiSIMS) for depth-resolved molecular profiling in tissue,^15^ complemented by DFT modeling to characterize supramolecular lipid interactions.

cryoSIM enables visualization of emissive organic dyes and metal complexes in vitrified cells, while SXT allows high-resolution, label-free assessment of organelle morphology. Applying both modalities to the same biological system can afford complementary insights into the localization of drugs and structural outcomes. Human A549 lung adenocarcinoma cells were treated with complex **1**, plunge-frozen, and imaged under cryogenic conditions at beamline B24 (Diamond Light Source) using cryoSIM for correlative analysis. In selected experiments, organelle-specific dyes provided additional subcellular context (Table S1). Surprisingly, complex **1** exhibited intense membrane-associated luminescence (Figure 2c), absent in untreated controls (Figure 2d). Figure 2f shows the dose-dependent intensity of the red fluorescence from the complex localized in the cell membranes.

**Figure 2:**
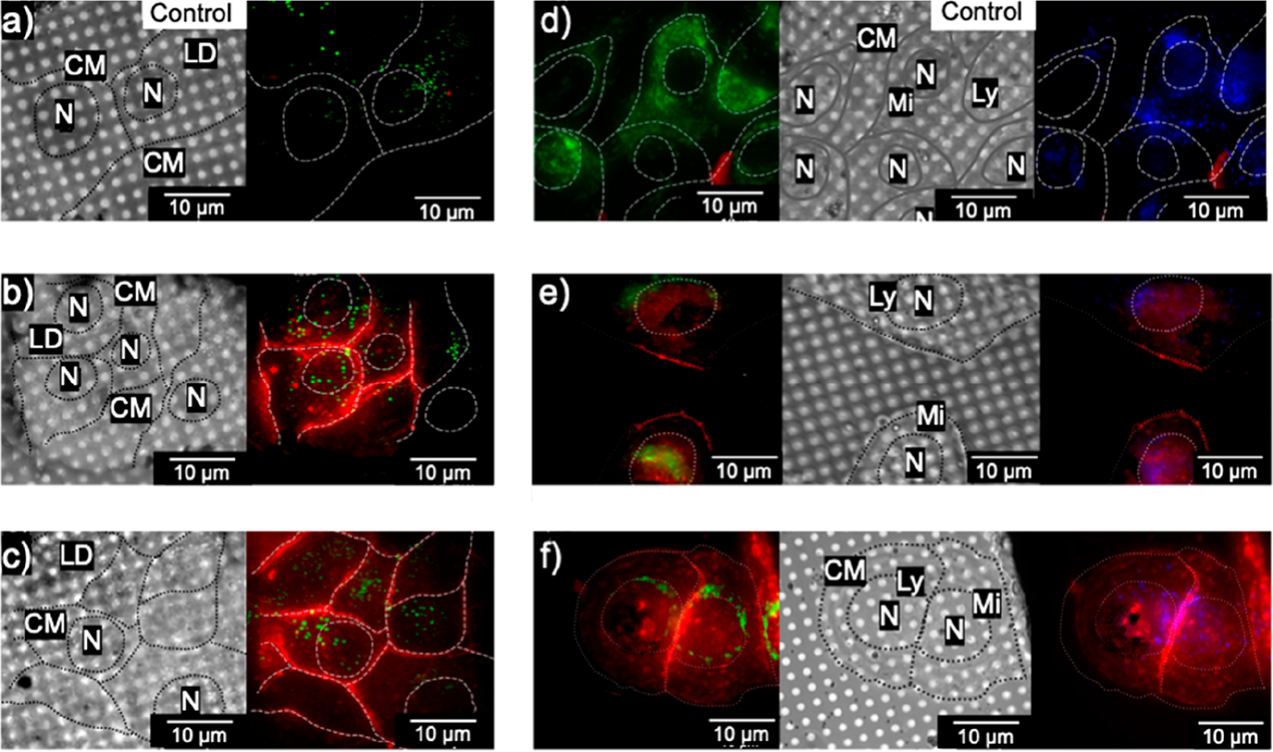
Correlated bright-field (left) and cryoSIM fluorescence (right) images of two representative areas of cryo-fixed human A549 lung cancer cells on Quantifoil carbon-gold grids. (a, d) Untreated (control) cells or (b, c) cells treated with 10 × IC_50_ **1** (300 μM, 1 h, 310 K in DMEM), or (e, f) 1 × IC_50_ **1** (30 μM, 1 h, 310 K in DMEM). Both treatment conditions show cell membrane localization. Stains: LipidSpot (λ_ex/em_ = 427/585 nm) (a, b, c), green emission; MitoTracker (λ_ex/em_ = 490/516 nm) (d, e, f), green emission; LysoTracker (λ_ex/em_ = 373/422 nm) (d, e, f), blue emission. Rh complex treatment (λ_ex/em_ = 561/605 nm), red emission. Lipid droplets, LD; nucleus, N; cell membrane, CM; lysosomes, Ly.

Strikingly, even at a high concentration of 300 μM (10 × IC_50_, 1 h treatment), **1** did not induce detectable structural damage, and treated cells retained morphology comparable to that of untreated controls (X-ray mosaics in Figure S1; cryoSXT in Movies S1 and S2; compare cryoSXT Movie S1 for control cells, with Movie S2 for treated cells). Consistent behavior was observed across independent experiments at IC_50_ concentration (30 μM), confirming a reproducible and selective interaction of **1** with lipid-rich membrane domains. While no major ultrastructural alterations were detectable during the first hour of exposure, the emergence of punctate red luminescence indicated localized accumulation of the complex, suggesting the initiation of early molecular events that may precede downstream cytotoxic responses occurring on longer time scales.

The downstream metabolic consequences were investigated by quantifying lipid droplet (LD) morphology. LDs play crucial roles in cell proliferation.^16^ They contain mostly triacyl glycerols, and also cholesteryl esters with embedded surface proteins including lipid droplet metabolism enzymes. Designs for luminescent metal complexes as stains for LDs have been reported, e.g., in refs 17 and 18.

Lipid droplet-rich fractions were isolated from A549 cells treated with complex **1**, and Rh content was determined by ICPMS (details in Section S2.15, Figure S2). Accumulation of Rh was ca. 3-fold higher at 5 × IC_50_ compared to 1 × IC_50_ (Figure S2). LipidSpot staining, widefield fluorescence imaging and FIJI analysis revealed significant enlargement of LDs in Rh­(III)-treated cells relative to controls, with increases in LD diameter of ca. 3.7-fold and volume of ca. 48-fold, but a ca. 4-fold decrease in LD number for cells treated with **1** (Figure 3d,e, SI Section S2.15). LDs can grow by acquiring lipids from the ER, or by fusing with other lipid droplets dependent on cell death-inducing DNA fragmentation factor-like effector (CIDE) proteins.^16^ The marked increase in droplet diameters and volumes induced by complex **1** are consistent with fusion or coalescence of smaller droplets into fewer, enlarged lipid stores. Notably the Rh species in the LDs did not give rise to detectable luminescence.

**Figure 3:**
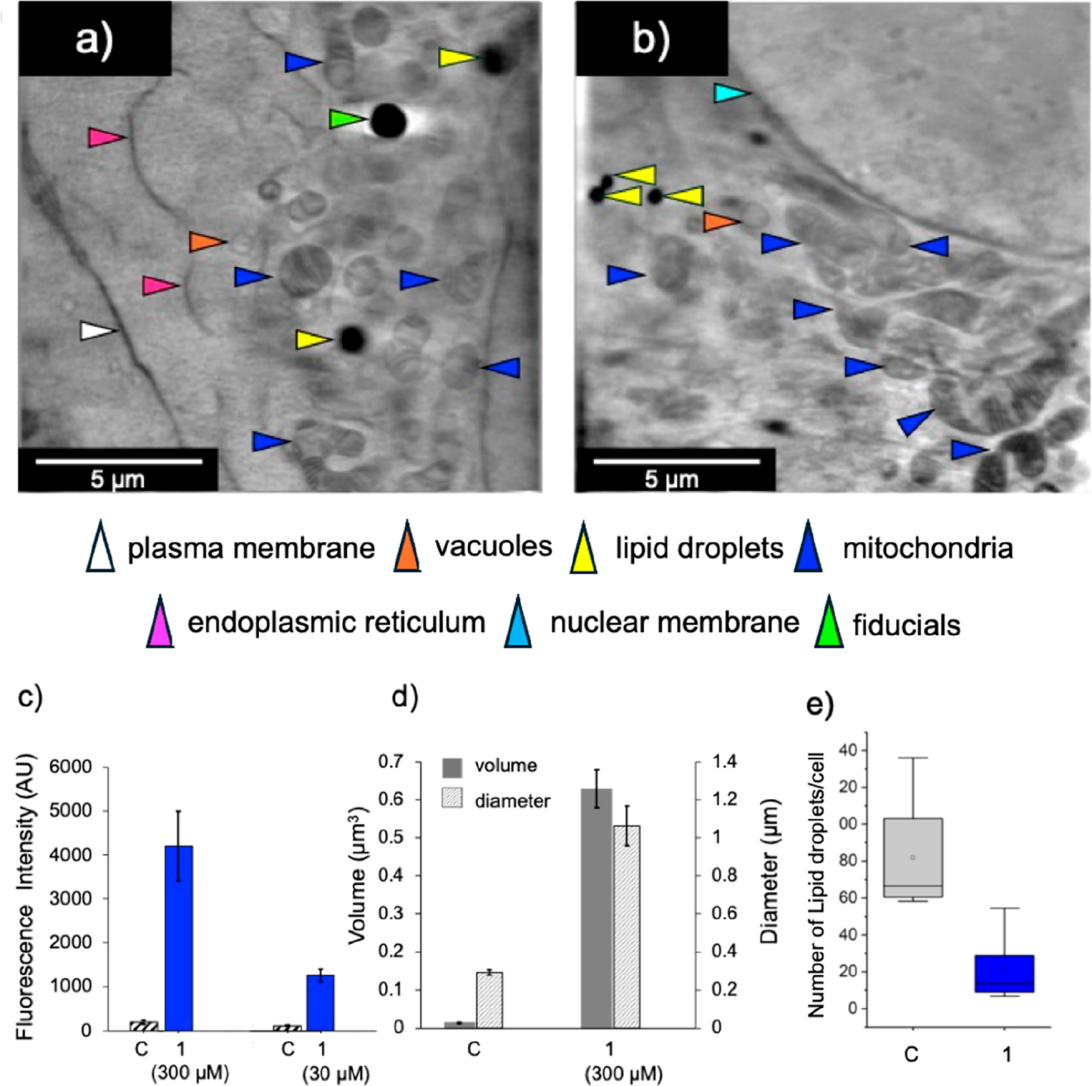
Effect of complex **1** on organelles in lung cancer cells. Soft X-ray tomograms of vitrified cells: (a) control cells and (b) cells after treatment with **1** (10 × IC_50_, 300 μM, 1 h), showing intact nuclei, mitochondria, lipid droplets, and ER, with the overall ultrastructure closely resembling that of untreated controls. (c) Quantification of red fluorescence in images from treated (*n* = 10 cells) and untreated (*n* = 59 cells) cells, across multiple independent grids. (d) Quantification of LD volume and diameter of treated and untreated cells. (e) Number of LDs after treatment with 300 μM **1**. Cells were stained with LipidSpot. Lipid droplets were segmented from LipidSpot-stained images and quantified for number, diameter, and volume with consistent thresholding across conditions, significant ca. 3.7-fold increase in mean diameter relative to controls. 3D cryoSIM reconstructions showed a ca. 48-fold increase in average droplet volume.

Remarkably, for a cytotoxic anticancer compound, cryogenic soft X-ray studies on vitrified A549 cells treated with complex **1** (10 × IC_50_, 300 μM) revealed that their ultrastructure is almost indistinguishable from controls. Tomogram reconstructions clearly resolved the nucleus, mitochondria, lipid droplets, endosomes, and endoplasmic reticulum, with little evidence of rupture or organelle-loss. Across multiple preparations, **1**-treated cells consistently preserved intact architecture suitable for semiquantitative analysis. The luminescence observed for complex **1** in cells was unexpected. Luminescent half-sandwich Rh­(III) complexes are rare.^18^ Typically they lack emission due to thermally accessible metal-centered (MC) states.^19^ TD-DFT calculations on **1** (gas phase) showed that the S_1_ state is primarily π → π* on the Me_2_N-azpy unit with ≤30% MLCT character (Figure 4a,b). Simulated emission at ∼650 nm matches the experimental maximum, supporting azopyridine as the emissive center (Figures S3–S6). While parent phenylazopyridine is nonfluorescent,^20^ substituents such as NMe_2_ in **1** can influence electron donation to the N=N bond. The stereochemistry of the N=N bond can have a major effect on its chemical and optical properties.^21^ For the free ligands, photoisomerization from the ground-state *trans* to excited state *cis* N=N configuration plays a role in the emission. The CNNC dihedral angle modulates the S_0_–S_1_ gap, impacting emission. Free ligands undergo *trans*–*cis* photoisomerization, but in complex **1**, the azo bond is locked in the *trans* (E) configuration, which stabilizes the emission behavior.

**Figure 4:**
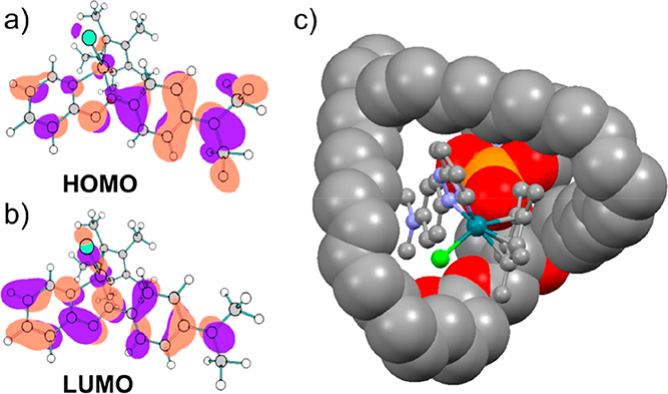
Electronic states of complex **1** and lipid binding. (a) HOMO and (b) LUMO from TD-DFT calculations (ωB97X-D3 functional). (c) DFT model of supramolecular adduct with glycer­ophos­phoryl­choline (spacefill) wrapped around **1** (ball and stick). Rh, turquoise.

Interactions of **1** with the model membrane phospholipids dioleoylphosphatidylcholine (DOPC, Figures 4c, and S7; solvent growth: Movies S3 and S4) and dilauroylphosphatidylcholine (DLPC, Figure S8) were explored by DFT modeling For DLPC, in a hydrophilic model (Figure S8a), the lipid quaternary ammonium substituent interacts with the chlorido ligand of **1** (N–Cl = 4.96 Å), and phosphate oxygens contact C3/C4 pyridine protons (2.17 and 2.53 Å). Together with lipid chain interactions, this results in a formation energy of 50 kJ/mol. In contrast, in the hydrophobic model (Figure S8b), the lipid chains wrap around Cp* and azopyridine rings, giving a more favorable formation energy of 70 kJ/mol. The DOPC supramolecular complex was even more stable (119 kJ/mol), with lipid chains nearly in contact around the Rh complex (C15–C17 = 4.3 Å) and a phosphate group near the Me_2_ *N*-phenyl unit (C4–PO = 3.8 Å; PO–N = 3.73 Å, Figure S7).

We investigated the penetration of complex **1** into veal brain homogenate (VBH) as a lipid-rich surrogate that mirrors the structural complexity and fluidity of native tissue, using OrbiSIMS. This combines the depth spatial precision of SIMS (<10 nm) with the ultrahigh mass resolution and accuracy of Orbitrap detection (*R* = 240,000), an even more powerful combination than time-of-flight (ToF)-SIMS.^22^ This can provide unambiguous identification of intact metal complexes, their fragments, and endogenous biomolecules.^23^

A solution of **1** deposited onto veal brain homogenate on a silicon wafer was dried, and analyzed using a 20 keV Ar^+^ cluster ion beam optimized to minimize surface damage while preserving molecular ion signal.^24^ Depth-profiling revealed persistent and colocalized signals for both [Rh]^+^ and intact **1** across the full tissue homogenate thickness, sectioned with 10 μm thickness (Figures 5, S9, and S10, Table S2). Additional signals for choline-containing lipids (VBH, [C_5_H_14_NO]^+^, *m*/*z* 104.1070) (Table S3) and the underlying silicon substrate ([Si_6_]^+^) served as depth benchmarks. Sputter-yield volumes were calibrated using an organic standard (Irganox).^25^

**Figure 5:**
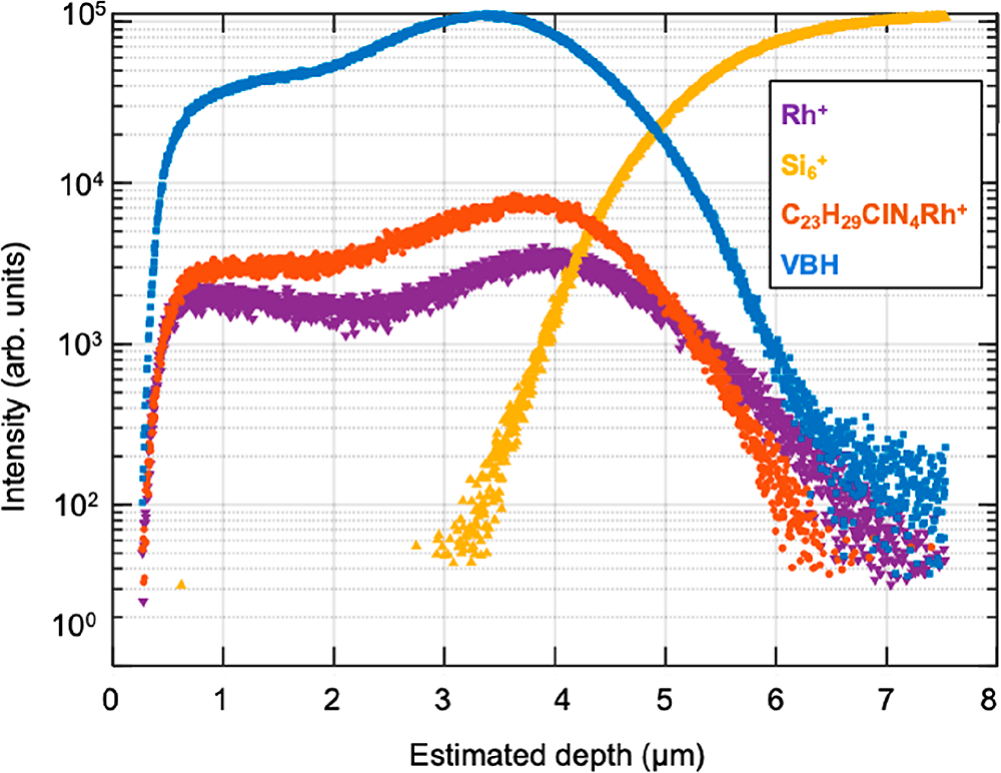
Penetration of complex **1** into a model tissue (VBH). OrbiSIMS positive-ion depth profiles obtained using a 20 keV Ar_2200_^+^ ion beam. Complex **1**, orange; [Rh]^+^, purple; [Si_6_]^+^, yellow; and [VBH], blue, where [VBH] is the sum of ion fragments characteristic of choline headgroups of phosphorylcholine lipids: [C_5_H_15_PNO_4_]^+^, [C_5_H_14_NO]^+^, and [C_5_H_12_N]^+^.

Ion dose–response analysis showed that the intensity of these ions changes as the sample is increasingly sputtered. Following an initial transient region attributed to stabilization of the surface charge, fragment Rh^+^ and **1** ions were detected at a high-intensity throughout the homogenate identified by the choline headgroup ions. At the interface with the silicon wafer their intensities fall sharply with a concomitant rise in Si_6_^+^. The similar ion intensity depth profiles for **1** and the VBH marker ions indicate that **1** is uniformly distributed throughout the depth of the homogenate tissue. Importantly, intact **1** was consistently detected across the homogenate depth, indicating that the complex remains chemically stable and structurally intact under these biologically relevant, lipid-rich conditions.

This work has revealed the multitargeting of a chelated half-sandwich Rh­(III) complex to lipid membranes and lipid droplets in human lung cancer cells, and provided insights into its strong molecular lipid interactions. Lipids represent underexplored targets for metallo-anticancer drugs. We envisage that machine learning incorporating the type of data reported here will eventually pave the way for the application of artificial intelligence to aid metallodrug design as it has done for targeted organic drugs.^26^ However, the complexity of both the thermodynamics and kinetics of metallodrug activation and speciation are formidable concepts for current AI technology.

## Supplementary Material

















## Data Availability

All data underlying this study are openly available for download, free of charge, as noted in the SI for cryoSIM and SXT in Section S2.10 and for OrbiSIMS in Section S2.13.

## Notes

Materials, experimental procedures, instruments and methods, fluorescence data, OrbiSIMS data, X-ray mosaic images, Rh content, DFT calculations and models, and TD-DFT absorption spectra (PDF)
DLPC_hydrophilic_interaction.pdb: DFT model of hydrophilic interaction of DLPC and **1** (PDB)
DLPC_hydrophobic_interaction.pdb: DFT model of hydrophobic interaction of DLPC and **1** (PDB)
DOPC_hydrophobic_interaction.pdb: DFT model of DOPC wrapped around **1** (PDB)
Movie S1: cryoSXT of control cells (AVI)
Movie S2: cryoSXT of treated cells (AVI)
Movie S3: DFT of solvent growth for **1** without lipid (MP4)
Movie S4: DFT of solvent growth for **1** with lipid (MP4)

## Abbreviations

cryoSXT: cryogenic soft X-ray tomography
cryoSIM: super-resolution fluorescence structured illumination microscopy (at cryogenic temperatures)
DFT: density functional theory
DOPC: diol­eoyl­phos­phat­idyl­choline
DLPC: dil­aur­oyl­phos­phat­idyl­choline
ESI-MS: electrospray ionization mass spectrometry
ICPMS: inductively coupled plasma mass spectrometry
OrbiSIMS: secondary ion mass spectrometry with Orbitrap detection
TD-DFT: time-dependent density functional theory
ToF-SIMS: time-of-flight secondary ion mass spectrometry

## References

[1] Mjos K. D., Orvig C. (2014). Metallodrugs in Medicinal Inorganic Chemistry. Chem. Rev..

[2] Gasser G., Ott I., Metzler-Nolte N. (2011). Organometallic Anticancer Compounds. J. Med. Chem..

[3] Casini A., Pöthig A. (2024). Metals in
Cancer Research: Beyond Platinum Metallodrugs. ACS Cent. Sci..

[4] Xiong X., Liu L.-Y., Mao Z.-W., Zou T. (2022). Approaches
towards
Understanding the Mechanism-of-Action of Metallodrugs. Coord. Chem. Rev..

[5] Anthony E. J., Bolitho E. M., Bridgewater H. E., Carter O. W. L., Donnelly J. M., Imberti C., Lant E. C., Lermyte F., Needham R. J., Palau M., Sadler P. J., Shi H., Wang F.–X., Zhang W.–Y., Zhang Z. (2020). Metallodrugs Are Unique:
Opportunities and Challenges of Discovery and Development. Chem. Sci..

[6] Scalese G., Kostenkova K., Crans D. C., Gambino D. (2022). Metallomics and Other
Omics Approaches in Antiparasitic Metal-Based Drug Research. Curr. Opin. Chem. Biol..

[7] CMarchi R., Harkiolaki M., Sadler P. J. (2025). A Correlative X-ray Bioimaging Triad
for Metals in Biomedical Research. Chem. Biomed.
Imaging.

[8] Jia F., Zhao X., Zhao Y. (2023). Advancements
in ToF-SIMS Imaging
for Life Sciences. Front. Chem..

[9] Gorman B. L., Torti S. V., Torti F. M., Anderton C. R. (2024). Mass Spectrometry
Imaging of Metals in Tissues and Cells: Methods and Biological Applications. Biochim. Biophys. Acta, Gen. Subj..

[10] Zhou Y., Li H., Tse E., Sun H. (2024). Metal-Detection Based Techniques
and Their Applications in Metallobiology. Chem.
Sci..

[11] Wang H., Zhou Y., Xu X., Li H., Sun H. (2020). Metalloproteomics
in Conjunction with Other Omics for Uncovering the Mechanism of Action
of Metallodrugs: Mechanism-Driven New Therapy Development. Curr. Opin. Chem. Biol..

[12] Romero-Canelón I., Sadler P. J. (2015). Systems Approach
to Metal-Based Pharmacology. Proc. Natl. Acad.
Sci. U.S.A..

[13] Lant E. C., Needham R. J., Zhang Z., Coverdale J. P., Clarkson G. J., Bagley I., Dallmann R., Sadler P. J. (2025). Cyclopentadienyl
Half-Sandwich Rhodium­(III) Azopyridine Anticancer Complexes with Activity
Tuned by Ligand Substituents. ChemCatChem..

[14] Eastham, J. H. ; Patel, P. Phenazopyridine. In StatPearls; StatPearls Publishing, Treasure Island, FL, 2025. https://www.ncbi.nlm.nih.gov/books/NBK580545/ (accessed 2025-10-10).

[15] Passarelli M. K., Pirkl A., Moellers R., Grinfeld D., Kollmer F., Havelund R., Newman C. F., Marshall P. S., Arlinghaus H., Alexander M. R., West A. (2017). The 3D OrbiSIMS—Label-Free
Metabolic Imaging with Subcellular Lateral Resolution and High Mass-Resolving
Power. Nat. Methods.

[16] Bohnert M., Schrul B. (2024). Lipid droplets in health
and disease. FEBS letters.

[17] Bader C. A., Brooks R. D., Ng Y. S., Sorvina A., Werrett M. V., Wright P. J., Anwer A. G., Brooks D. A., Stagni S., Muzzioli S. (2014). Modulation
of the Organelle Specificity in
Re­(I) Tetrazolato Complexes Leads to Labeling of Lipid Droplets. RSC Adv..

[18] Lee L. C.-C., Lo K. K.-W. (2024). Shining New Light on Biological Systems: Luminescent
Transition Metal Complexes for Bioimaging and Biosensing Applications. Chem. Rev..

[19] Indelli, M. T. ; Chiorboli, C. ; Scandola, F. Photochemistry and Photophysics of Coordination Compounds: Rhodium. In Photochemistry and Photophysics of Coordination Compounds I; Springer, Berlin, Heidelberg, 2007; pp 215–255.

[20] Yoopensuk S., Tongying P., Hansongnern K., Pakawatchai C., Saithong S., Tantirungrotechai Y., Leesakul N. (2012). Photoactive Azoimine
Dyes: 4-(2-Pyridylazo)-N, N-Diethylaniline and 4-(2-Pyridylazo)-N,
N-Dimethylaniline: Computational and Experimental Investigation. Spectrochim Acta A Mol. Biomol. Spectrosc..

[21] Xu Y., Gao C., Andréasson J., Grøtli M. (2018). Synthesis
and Photophysical Characterization of Azoheteroarenes. Org. Lett..

[22] Trindade G., Paterson R., Yan B., Vorng J.-L., Pirkl A., Gilmore I. (2025). OrbiSIMS Spatially
Resolves Isomeric Molecules on Surfaces. Research
Square Preprint.

[23] Zhang J., Brown J., Scurr D. J., Bullen A., MacLellan-Gibson K., Williams P., Alexander M. R., Hardie K. R., Gilmore I. S., Rakowska P. D. (2020). Cryo-OrbiSIMS for 3D Molecular Imaging of a Bacterial
Biofilm in Its Native State. Anal. Chem..

[24] Matjacic L., Seah M. P., Trindade G. F., Pirkl A., Havelund R., Vorng J., Niehuis E., Gilmore I. S. (2022). OrbiSIMS Metrology
Part I: Optimisation of the Target Potential and Collision Cell Pressure. Surf. Interface Anal..

[25] Seah M. P., Havelund R., Gilmore I. S. (2016). Systematic
Temperature Effects in
the Argon Cluster Ion Sputter Depth Profiling of Organic Materials
Using Secondary Ion Mass Spectrometry. J. Am.
Soc. Mass Spectrom..

[26] Zhang K., Yang X., Wang Y., Yu Y., Huang N., Li G., Li X., Wu J. C., Yang S. (2025). Artificial intelligence
in drug development. Nat. Med..

