# Supplementary material for: In Situ Lipid Interactions of an Anticancer Metal Complex
Source: Inorg Chem. 2026 Mar 13;65(12):6367–72. doi: 10.1021/acs.inorgchem.6c00104 (PMC13040523; doi:10.1021/acs.inorgchem.6c00104)
Supplement: Supplementary file 1 [file ic6c00104_si_001.pdf]

Supporting Information

In Situ Lipid Interactions of an Anticancer Metal Complex

*Edward C. Lant,[a] Archana C. Jadhav,[b] Annabel Sumeray,[c] Gustavo F. Trindade,[c] Luca Craciunescu,[d] Andrew W. Prentice,[d] Juliusz A. Wolny,[e] Jaspreet S. Grewal,[f] Robert Dallmann,[f] Guy J. Clarkson,[a] Ann M. Dixon,[a] Volker Schünemann,[e] Ian S. Gilmore,[c] Martin J. Paterson,[d] Maria Harkiolaki,[a,b] Peter J. Sadler[a]**

[a] *Department of Chemistry, University of Warwick, UK*

[b] *Diamond Light Source,, UK*

[c] *National Physical Laboratory (NPL), UK*

[d] *School of Engineering & Physical Sciences, Heriot-Watt University, UK*

[e] *Department of Physics, University of Kaiserslautern-Landau, Germany*

[f] *Division of Biomedical Sciences, University of Warwick, UK*

*P.J.Sadler@warwick.ac.uk

Table of Contents

S1 Experimental S3
  S1.1 Materials S3
  S1.2 Synthesis and Characterisation S3
S2 Instruments and methods S3
  S2.1 DFT Computational methods S3
  S2.2 DFT models of supramolecular complex **1** and glycerophosphorylcholine lipids S3
  S2.3 Optical properties (TD-DFT). S4
  S2.4 X-ray crystallography. S4
  S2.5 Inductively coupled plasma-mass spectrometry (ICP-MS). S4
  S2.6 Cell Culture S5
  S2.7 Cryogenic fluorescence microscopy. S5
  S2.8 Cryo-Structured Illumination Microscopy (CryoSIM). S5
  S2.9 Cryo soft-X-ray tomography (CryoSXT). S5
  S2.10 Sample preparation for CryoSIM. S6
  S2.11 Sample preparation for CryoSXT. S6
  S2.12 Statistical analysis. S6
  S2.13 Orbitrap Secondary Ion Mass Spectrometry (OrbiSIMS). S7
  S2.14 Sample preparation for OrbiSIMS S8

S2.15 Lipid droplet investigation S8

Table S1. Quantification of fluorescence signals in A549 cells following 1 h incubation at-310 K S12
Table S2. Positive ion peaks characteristic of the choline headgroups of glycerophosphoryl lipids used to track VBH S13
Table S3. Peaks monitored in OrbiSIMS study of the penetration of **1** into VBH tissue.. .... S13
Figure S1. X-ray mosaic images of A549 human lung cancer cells a) control (untreated), b) treated with 10 x IC_{50} **1** (300 μM) for 1 h S14
Figure S2. Rh content in lipid droplets isolated from A549 cells after 1 h incubation with compound **1** S15
Figure S3. Solvent growth for **1** without lipid obtained with the QCG algorithm in the CREST program suite. S16
Figure S4. Solvent growth for **1** with lipid obtained with the QCG algorithm in the CREST program suite. S16
Figure S5. TD-DFT absorption spectra of complex **1** with and without lipid present obtained with the B3LYP functional.. .... S17
Figure S6. Absorption spectra of **1** in vacuum and water for various DFT functionals and ADC(2). S18
Figure S7. DFT model of DOPC lipid wrapped around **1**. S19
Figure S8. Optimised DFT showing (a) hydrophilic and (b) hydrophobic interaction of the lipid 1,2-dilauroyl-sn-glycero-3-phosphocholine (DLPC) and **1**. S20
Figure S9. Representative OrbiSIMS spectrum showing ions detected for a control silicon grid treated with complex **1** S21
Figure S10. Measurement of the penetration of **1** (20% MeOH/80% H_2O, v/v) in VBH using OrbiSIMS S21

S1 Experimental

S1.1 Materials. $[(Cp^*)Rh(\mu\text{-}Cl)Cl]_2$ was purchased from Sigma-Aldrich, 4-NMe_2-phenylazopyridine and ammonium hexafluorophosphate from Fisher Scientific, solvents including DMSO-d_6, MeOD-d_4, and D_2O, from Sigma-Aldrich, A549 cell line from ECACC (European Collection of Authenticated Cell Cultures). All reagents were used as received unless specified.

S1.2 Synthesis and Characterisation. Complex **1** was synthesised as a PF_6 salt as previously described,[3] with satisfactory ^1H and ^{13}C NMR and HR-MS characterization, and an HPLC purity of >97%. A single crystal suitable for X-ray diffraction analysis was obtained by slow diffusion of diethyl ether into a concentrated ethanol solution of the complex over several days at ambient temperature.

S2.0 Instruments and methods

S2.1 DFT Computational methods. DFT calculations for the lipid-complex adducts were performed with Gaussian 16[4] using B3LYP functional[5] and cep-31g[6–8] basis set with Grimme's D3[9] dispersion correction. Water was modelled as solvent using the IEFPCM approach.

S2.2 DFT models of supramolecular complexes of 1 and glycerophosphoryl-choline lipids. Structures of lipid 1,2-dioleoyl-sn-glycero-3-phosphocholine (DOPC) and 1,2-dilauroyl-sn-glycero-3-phosphocholine (DLPC) examined in this study are in Fig.1 c/d. For the lipid DLPC, two binding modes with complex **1** were considered based on DFT-optimized structures. The first mode involves predominantly hydrophilic interactions (Fig. S8), where the quaternary ammonium group of DLPC orients toward the chlorido ligand of **1** (N···Cl = 4.9 Å). Additionally, the phosphate oxygens engage in close contacts with the C3 and C4 pyridyl protons (2.22 and 2.47 Å, respectively), Fig. S8. No significant interactions were observed between the lipid tails and the complex. The calculated formation energy for this assembly is 41 $kJ \cdot mol^{-1}$.

In contrast, the second mode is driven by hydrophobic interactions (Figure S8), wherein the lipid chains envelop the complex, effectively encapsulating **1** within the lipid matrix. This configuration yields a significantly more favourable formation energy

of 69 kJ·mol^{-1}, indicating a stronger affinity via hydrophobic association. Optimized structures for both binding modes are shown in Fig. S8.

S2.3 Optical properties (TD-DFT). Modelling of explicit solvent molecules was carried out with the QCG algorithm[10] in the CREST[11] and xtb[12,13] program packages. TD-DFT calculations were carried out with the Orca 5.0.3[14–16] (or Gaussian 16 Revision C.02)[4] electronic structure suites. All DFT calculations made use of the def2-TZVP basis set for Rh and def2-SVP on all other atoms.[17] To understand how lipid molecules influence the interaction between **1** and solvent, in this case water, solvent cluster growth simulations were carried out. In these calculations the lipid molecule DOPC was found to shield the complex effectively from water molecules, as the total number of water molecules for the converged solute-solvent interaction was 19 for the complex wrapped in lipid and 35 for the complex on its own. These findings lead to the feasible assumption that for description of excited states, no polarizable continuum model has to be used, as the transition metal complex effectively does not sense the solvent. Therefore, TD-DFT calculations were carried out in vacuum.

S2.4 X-ray crystallography. A suitable crystal of **1** was selected and mounted on a glass fiber with Fomblin oil and placed on a Rigaku Oxford Diffraction SuperNova diffractometer with a dual source (Cu at zero) equipped with an AtlasS2 CCD area detector. The crystal was kept at 150 ± 2 K, during data collection. Using Olex2,[18] The structure was solved with the ShelXT[19] structure solution program using direct methods and refined with the ShelX[20] refinement package using least squares minimization. X-ray crystallographic data for complex **1** have been deposited in the Cambridge Crystallographic Data Centre under the accession number CCDC 2454589. X-ray crystallographic data in CIF format are available from the Cambridge Crystallographic Data Centre (http://www.ccdc.cam.ac.uk/). The data were processed using Mercury 4.0. software.[21]

S2.5 Inductively coupled plasma-mass spectrometry (ICP-MS). ICP-MS analysis was carried out using an Agilent Technologies 7500 series ICP-MS. All samples and standards were prepared on the day of analysis in nitric acid (3.6% *v*/*v*, using freshly distilled nitric acid and doubly deionised water). The rhodium CRM standard was diluted to achieve a working concentration range of 0.1-100 ppb. Samples were diluted by serial dilution until their concentrations were within the calibration range and the %

total dissolved solids was below 0.1% *w/v.* Data were acquired and processed using Agilent ChemStation for Windows (7500 series ICP-MS) or Agilent Mass Hunter 4.3 for Windows (7500 series ICP-MS). Biological triplicates were produced for each sample and statistical significance was calculated (Welch's unpaired t-test).

S2.6 Cell Culture. Cancer cells were grown in Dulbecco's Modified Eagle Medium (DMEM, phenol red-free) supplemented with 10% v/v foetal calf serum (FCS), 1% v/v penicillin streptomycin antibiotics and 1% v/v L-glutamine (2 mM) and warmed to 310 K prior to use. Cells were grown in T-75 or T-175 culture flasks as adherent monolayers at 310 K with a 5% CO_2 humidified atmosphere. Cells were passaged 2-3 times per week whenever confluence reached 80-90% using 0.25% trypsin/EDTA for all other cell lines. Cells were used between 5-25 passages.

S2.7 Cryogenic fluorescence microscopy. Correlative microscopy analysis of plunge-frozen Quantifoil carbon-gold TEM grids was performed on a Zeiss Axioimager M2 (Carl Zeiss X-ray Microscope) coupled to a Linkam cryostage (CMS196M LED) for preliminary grid analysis and mapping (max resolution 50x, variable filters) by monitoring the fluorescence of trackers used.

S2.8 Cryo-Structured Illumination Microscopy (CryoSIM). Prior to cryo-soft X-ray tomography (cryoXRT), super-resolution fluorescence microscopy was performed on plunge-frozen TEM grids using the cryo-SIM facilities (B24 beamline, DLS). The cryoSIM has 4 excitation lasers (405, 488, 561 and 642nm; 10-100 Wcm^{-2} laser power) and a 100X long distance air objective (2 mm, 0.9NA), and can achieve a lateral resolution of 360 nm (when using green light, λ_{ex} 488 nm).[22,23]

S2.9 Cryo soft-X-ray tomography (CryoSXT). The cryo-SXT and super-resolution cryoSIM measurements were performed at the full-field transmission microscope at beamline B24 using a Zeiss UltraXRM-S220C microscope end station.[23] The B24 beamline operates in the soft X-ray region (200-2600 eV), using a transmission microscope operating at 500 eV, achieving a resolution of 30-40 nm and delivering a photon flux 10^8 photons/μm.[22,24] Data were collected by direct detection using a CCD (Pixis, XO 1024B; Princeton Scientific).11 B24 uses a bending magnet source to generate X-rays, which are directed by a toroidal mirror[22,24] onto a Variable Line Spacing (VLS) grating monochromator. The mirror focuses the beam onto a focal point downstream of the monochromator, where a slit system removes stray radiation and

selects the desired wavelength for the experiment. The beam is then focussed onto the sample using a capillary condenser. Downstream from the sample, a zone plate (40 nm minimum spacing) projects the image onto the Charge Coupled Device (CCD) detector.

S2.10 Sample preparation for CryoSIM. A549 cells were cultured as described above, and incubated with complex **1** (solubilized in DMEM-phenol red-free/DMSO <5%), with added fluorophores: (i) MitoTracker™ Green ($\lambda_{ex/em}$= 490/516 nm), LysoTracker™ Blue ($\lambda_{ex/em}$= 373/422 nm) and LipidSpot™ Green ($\lambda_{ex/em}$= 427/585 nm). Cells were plunge-frozen in liquid ethane (185 K) and the fluorophores monitored by fluorescence microscopy on a Zeiss Axioimager M2 microscope coupled to a cryo-stage (model CMS196M, Linkam Scientific, UK) to identify suitable cell candidates for analysis by cryoXRT.[25] CryoSIM studies were undertaken on frozen-hydrated A549 cells grown on carbon–gold TEM grids and treated with 10 x IC_{50} (300 μM) and 1 x IC_{50} (30 μM) complex **1.**

S2.11 Sample preparation for CryoSXT. The plunge-frozen grids used for cryoSIM were loaded into the sample chamber of the beamline. X-ray mosaic images were obtained first using short exposures (0.5 s). Regions of interest were brought into focus by mapping the positions of gold fiducials and dense lipid droplets in the tilt range − 30° and + 30°. The image focus was adjusted by altering the position of the 40 nm zone plate objective. Tomograms were acquired with a tilt range of -65° to +65° using 0.2° - 0.5° rotations and 1-8 s/frame X-ray exposure.

The tomograms for control cells and for cells treated with 300 μM **1** are shown in Movies S1 (Movie_S1_cryoSXT-Control) and S2 (Movie_S2_cryoSXT-Treated).

CryoSIM and SXT data deposition. The B24 beamline at Diamond Light Source has a published workflow for depositing correlative data collected using cryo-SIM and cryo-SXT microscopes into the BioStudies/EMPIAR archive (https://doi.org/10.1016/j.xpro.2020.100253).

S2.12 Statistical analysis. Results are presented as mean ± standard deviation (SD) unless otherwise stated. Statistical significance was determined using a Welch's t-test at the 95% confidence level. Correlation analyses were performed using Pearson's

correlation coefficient, and coefficient of determination (R^2) values were reported to indicate the goodness of fit for linear regression models.

S2.13 Orbitrap Secondary Ion Mass Spectrometry (OrbiSIMS). SIMS spectra, profiles and images were acquired using an OrbiSIMS (Hybrid SIMS, IONTOF GmbH, Muenster, Germany), which is a dual analyser SIMS instrument incorporating an Orbitrap™ mass analyser (Thermo Fisher Scientific, Bremen, Germany) and a time-of-flight (ToF) mass analyser.[8] All samples were mounted onto the OrbiSIMS top-mount holder and introduced into the load lock of the instrument. Once a stable vacuum was established, samples were moved into the main chamber for analysis.

All analyses were conducted with the Orbitrap mass spectrometer with an injection time of 500 ms resulting in a mass-resolving power of 240,000 at *m*/*z* 200. Low energy electron flooding and argon gas regulation were used for charge compensation.

Calibration of the Orbitrap analyser was performed using a Bi_3^+ liquid metal ion gun on a silver sample, using silver cluster secondary ions and the method performed by Passarelli et al.[26] For all analyses, the He cell was set to "low collisional cooling" with a He pressure of 4.7×10^{-2} mbar. Mass spectra were acquired in positive and negative ion polarity using a 20 keV Ar_{2200}^+ primary ion beam with a spot size of ca. 20 µm diameter from an area of 400 × 400 µm using random raster mode. Depth profiles were obtained in a single beam mode using a 20 keV Ar_{2500}^+ (GCIB) as the primary ion beam using random raster mode.[27] Secondary ions were collected from an area of 200 µm × 200 µm. Depth profiles were collected in positive ion polarity for 2000 scans (2000s sputter time) with a mass range *m*/*z* 80–1200. For sample **1**, the GCIB duty cycle was 15% and 20% respectively delivering 1 nA and 1.5 nA and the surface potential was set respectively at 18 V and 24.5 V. The pressure in the main chamber was maintained at 9.6×10^{-7} bar with the argon gas regulation on. IONTOF SurfaceLab 7.5 was used to acquire the data and process the results and assign the peaks. An estimation of the real depth of **1** in VBH tissue was made using measured sputter yield volumes of an organic molecular standard (Irganox)[28]. The Ar_{2200}^+ primary ion beam for minimises damage to the soft tissue and preserves the chemical integrity of molecular fragments. A summary of peaks is in Table S2.

All OrbiSIMS data reported here are available as imzML, ASCII and MATLAB .fig files. imzML files can be processed in MATLAB using the imzML parser of SpectralAnalysis (https://github.com/AlanRace/SpectralAnalysis).

S2.14 Sample preparation for OrbiSIMS. Seven 2 x 2 cm (0.5 mm thick) silicon wafers were cut using a Diamond Tipped Plastic Scribe and cleaned with compressed air and 2-propanol. They were then stored at room temperature until needed. Veal brain homogenates (VBH) were sectioned sequentially to 10 µm thickness using a Thermo CryoStar NX70 (Thermo Scientific) with -20°C sample holder and -20°C knife temperature. Five sections were thaw-mounted onto five of the pre-cut silicon wafers. Samples were vacuum sealed using a Henkelman Boxer 42 Packer (Henkelman UK) and stored at -80 °C until required for sample deposition. The samples were left to defrost at room temperature for 30 min prior to depositing the Rh complexes.1 µL drops of **1** dissolved in MeOH/H_2O 80%/20% vol/vol were micro pipetted and dropped onto two of the Si wafers with VBH. The two Si wafers left without any VBH had 1 µL of **1** dropped on the wafer to be used as positive controls; representative MS for positive control shown in Fig. S9. After all data were acquired, samples were removed from the instrument, vacuum sealed, and stored at -80 °C.

S2.15 Lipid droplet investigation

To investigate potential metabolic consequences of complex **1** exposure, changes in lipid droplet morphology of A549 cells were quantified after treatment (300 µM, 1 h, 310 K). Following LipidSpot™ staining, cells were imaged by fluorescence microscopy, and droplet diameter, volume, and number were quantified from maximum intensity projections using FIJI (ImageJ).

Fluorescence Intensity Quantification. A549 cells were seeded in phenol red–free DMEM and treated with Rh(III) complex **1** at 10xIC_{50} concentration (300 µM) for 1 h at 310 K. Cells were fixed and imaged using widefield fluorescence microscopy with consistent acquisition parameters. Z-stacks were reduced to maximum intensity projections. Subcellular regions of interest were manually drawn in FIJI, and background subtraction was applied using a rolling ball radius of 50 pixels. Corrected fluorescence intensities were extracted and expressed in arbitrary units (AU). At least 17 cells per treatment group were analyzed (n = 17-59). These data are semi-quantitative and intended for relative comparison rather than absolute quantification.

A one-way ANOVA across all four groups revealed a statistically significant difference in lipid-associated fluorescence ($F(3, 12) = 238.68$, $p < 0.001$). While fluorescence intensity was consistent across the two control groups, both -treated groups exhibited markedly elevated levels.

Lipid Droplet Size Analysis. LipidSpot™ 488 (PerkinElmer) staining was performed following compound treatment under identical conditions to the fluorescence assay. Cells were imaged by widefield fluorescence microscopy using fixed acquisition settings. Z-stack images were acquired and processed as maximum intensity projections prior to quantification and background-corrected with a rolling ball radius of 50 pixels in FIJI. Lipid droplets were segmented using automatic thresholding (Triangle or Otsu method), and droplet size was quantified using the Analyze Particles function with a minimum threshold of 0.1 µm. The mean droplet size per cell was calculated and normalized to the control group. Values reflect biological replicates (n = same cells used for fluorescence analysis) and represent semi-conservative size estimates sufficient for relative comparison.

Lipid Droplet Diameter Quantification Using 3D CryoSIM Imaging

Statistical Analysis. Lipid droplet diameters were compared between complex **1** and control using a two-sample t-test assuming unequal variances (Welch's t-test). To account for the differing number of cells measured per image, a weighted analysis was performed, expanding each measured droplet diameter according to the number of cells analyzed in that field. The resulting analysis yielded a t-statistic of 3.04 and a *p*-value << 0.001, indicating a highly significant increase in droplet diameter in complex **1**-treated cells relative to control.

The control condition consistently exhibited the lowest values, reinforcing the specificity and directionality of treatment-dependent changes. This analysis of from selective data and the whole population provides a semi-quantitative framework for interpreting treatment efficacy, highlighting meaningful trends in the dataset even with moderate sample sizes and inherent biological variability.

Lipid Droplet Volume Quantification Using 3D CryoSIM Imaging

Lipid droplet volumes were quantified using three-dimensional super-resolution cryogenic structured illumination microscopy (CryoSIM) at beamline B24 of the Diamond Light Source (Harwell, UK) (Fig. 3). Reconstructed image stacks were imported into FIJI (ImageJ), converted to 8-bit format, and subjected to background subtraction using a rolling ball radius of 50 pixels. Thresholding was performed using Otsu's method, selected for its robustness in separating foreground lipid signal from background under varying intensity conditions. Following thresholding, lipid droplets were segmented using the 3D Objects Counter plugin. Voxel dimensions were calibrated according to CryoSIM pixel scaling to extract absolute volume measurements for each segmented object.

To ensure data integrity, only droplets exceeding a volume threshold of 0.05 μm^3 were included in the analysis. Total lipid volume per cell was calculated by summing all segmented droplet volumes within a given field. A minimum of 11 cells (control, n = 11), and 59 cells for complex **1** (n = 59).

Lipid droplet volumes were compared between complex **1** and control using a two-sample t-test assuming unequal variances (Welch's $t = 37.74$, $p << 0.001$)). To account for differing numbers of cells measured per image, a weighted analysis was performed by expanding each measured volume according to the number of cells analyzed in that field.

Lipid Droplet Number Analysis

Lipid droplet numbers per cell were compared between complex **1** and control using a two-sample t-test assuming unequal variances (Welch's t-test). To account for differing numbers of cells measured per area, a weighted analysis was performed by expanding each measured droplet count according to the number of cells in that area, calculating droplets per cell. This analysis revealed a significant reduction in lipid droplet number for complex **1**-treated cells compared to control. Weighted analysis yielded mean droplet numbers of 13.4 ± 10.8 per cell for **1** (n = 59 cells) and 20.6 ± 3.8 per cell for control (n = 17 cells), corresponding to a t-statistic of –4.33 and a p-value of ($p < 0.001$). These results indicate that complex **1** promotes a morphological shift toward larger, less numerous lipid droplets.

Rh Accumulation in Lipid Droplets

Based on B24 CryoSIM studies suggesting these organorhodium complexes alter lipid droplet morphology, including increased droplet diameter and volume, a lipid droplet isolation study was undertaken to determine if they accumulate Rh. Lipid droplets, the primary site of neutral lipid storage, are dynamic organelles involved in metabolic signalling, oxidative stress responses, and cancer progression.

A549 cells were treated with **1** at concentrations of 1 × IC_{50} and 10 × IC_{50} for 24 h at 310 K in complete DMEM supplemented with ~1% DMSO. Following treatment, a multi-step fractionation protocol was employed to isolate lipid droplet–enriched layers. The fractions analysed included: (1) lipid droplet fraction obtained by flotation following cell lysis, (2) the remaining cell pellet after centrifugation at 1000 × *g* for 15 min, (3) untreated control samples (DMEM with 5% DMSO), and (4) whole-cell lysates used to assess total Rh content.

Rhodium levels in each fraction were quantified by inductively coupled plasma-mass spectrometry (ICP-MS). Notably, significant accumulation of Rh was detected in the lipid droplet–enriched fraction following treatment with **1**, as shown in Fig.S2. These findings suggest a potential organelle-targeting behaviour.

The accumulation of Rh in the different cell fractions treated with complex **1** increased in a concentration-dependent manner (Fig. S2). After 24 h the cellular Rh accumulation of **1** in lipid droplets was *ca.* 3-fold at 5 x IC_{50} than at 1 x IC_{50}. Even at low concentrations of **1** there was a significant accumulation of Rh inside the lipid droplets.

Tables

Table S1. Quantification of fluorescence signals from A549 cells following 1 h incubation at 310 K. Cells treated with Lipid Spot (LS) or with MitoTracker and LysoTracker staining (MT+LT), at 10 × IC_{50} and 1 × IC_{50}, respectively (see Fig 2). Data include the total number of cells in X-ray mosaics, total number of mosaics analysed, and number of fluorescent cells per area.

Treatment	Condition	No. of cells fluorescent/ area	Total no. X-ray mosaics	Total cells in X-ray mosaics
**1** (+LS)	10 × IC_{50}	59	9	59
control (+LS)	untreated	-	4	17
**1** (+MT+LT)	1 × IC_{50}	10	5	10
control(+MT+LT)	untreated	-	3	17

Table S2. Peaks monitored in OrbiSIMS study of the penetration of **1** into VBH tissue (Fig. 5).

m/z	Assignment	Mass Deviation (ppm)	Component
102.9050	$[Rh]^+$	0.0	Fragment from 1
167.8610	$[Si_6]^{+\ b}$	0.2	$[Si_6]^+$ (Si wafer)
463.1441	$[C_{23}H_{28}N_4Rh]^+$	-0.1	$[1\text{-}HCl]^+$
499.1131	$[C_{23}H_{29}ClN_4Rh]^+$	0.1	$[1]^+$

Table S3. Positive ion peaks characteristic of the choline headgroups of glycerophosphoryl lipids used for tracking in OrbiSIMS depth profiling analysis of VBH tissues (see Fig 5).

m/z	Assignment	Mass deviation/ppm
184.0734	$C_5H_{15}PNO_4^+$	0.4
104.1069	$C_5H_{14}NO^+$	-0.3
86.0967	$C_5H_{12}N^+$	2.7

Figures

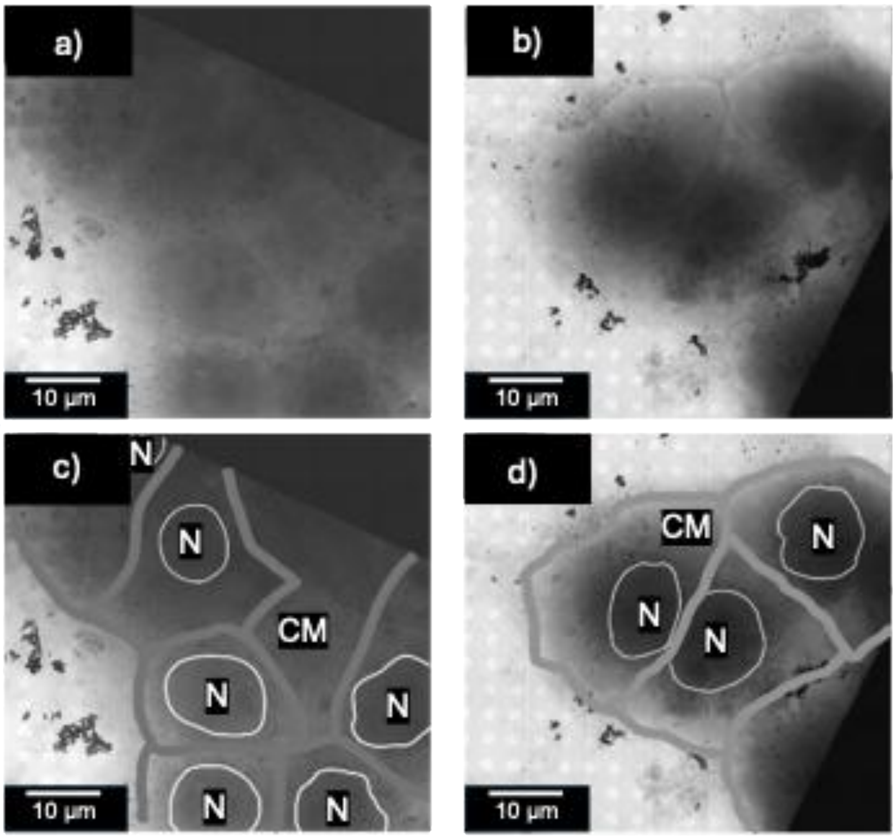

Figure S1. X-ray mosaic images of A549 human lung cancer cells; a) control (untreated), b) treated with 10 x IC_{50} **1** (300 µM) for 1 h. c) and d) are marked with overlays showing the distinct observable features identified in a) and b) respectively: nucleus (N), cell membrane (CM).

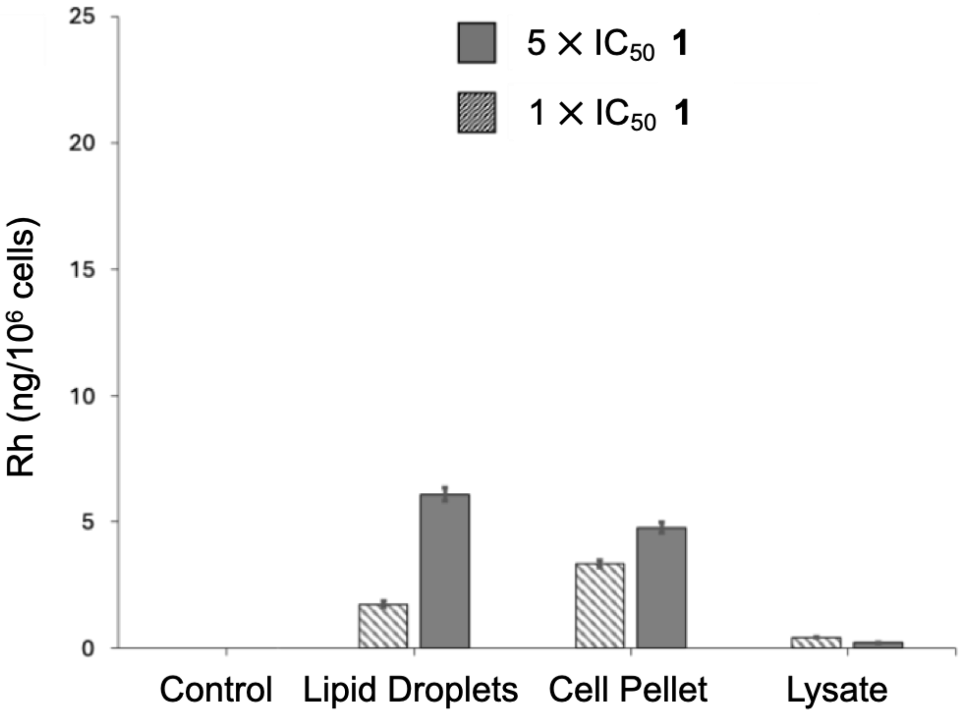

Figure S2. Rh content of lipid droplets, cell pellet and lysate isolated from A549 cells after 1 h incubation with complex **1**, as determined by ICP–MS. Control corresponds to lipid droplets from untreated cells.

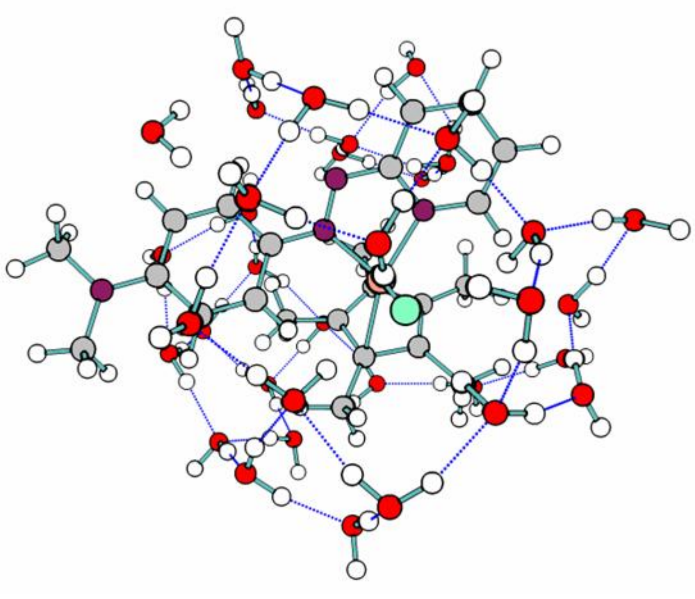

Figure S3. Solvent growth for **1** without lipid obtained by DFT calculations using the QCG algorithm in the CREST program suite. See Movie_S3_DFT_solvent growth_nolipid.mp4

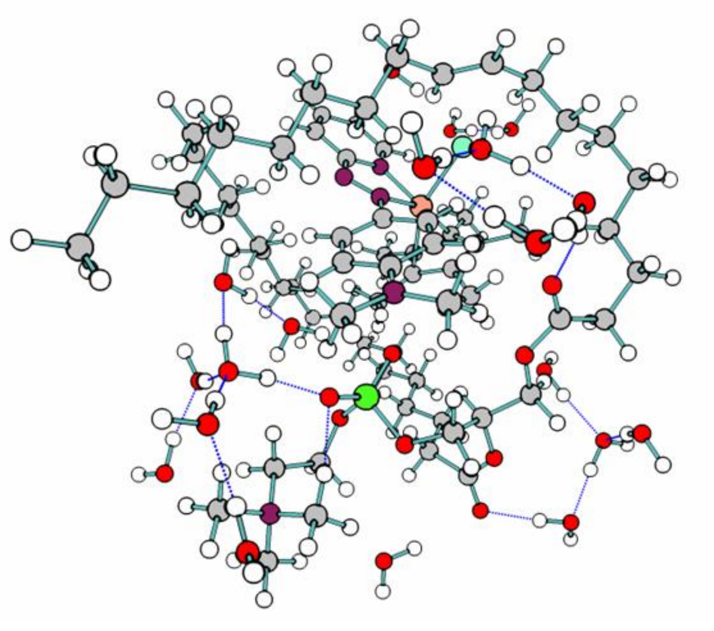

Figure S4. Solvent growth for **1** with lipid obtained by DFT calculations with the QCG algorithm in the CREST program suite. See Movie_S4_DFT_solvent growth_lipid.mp4.

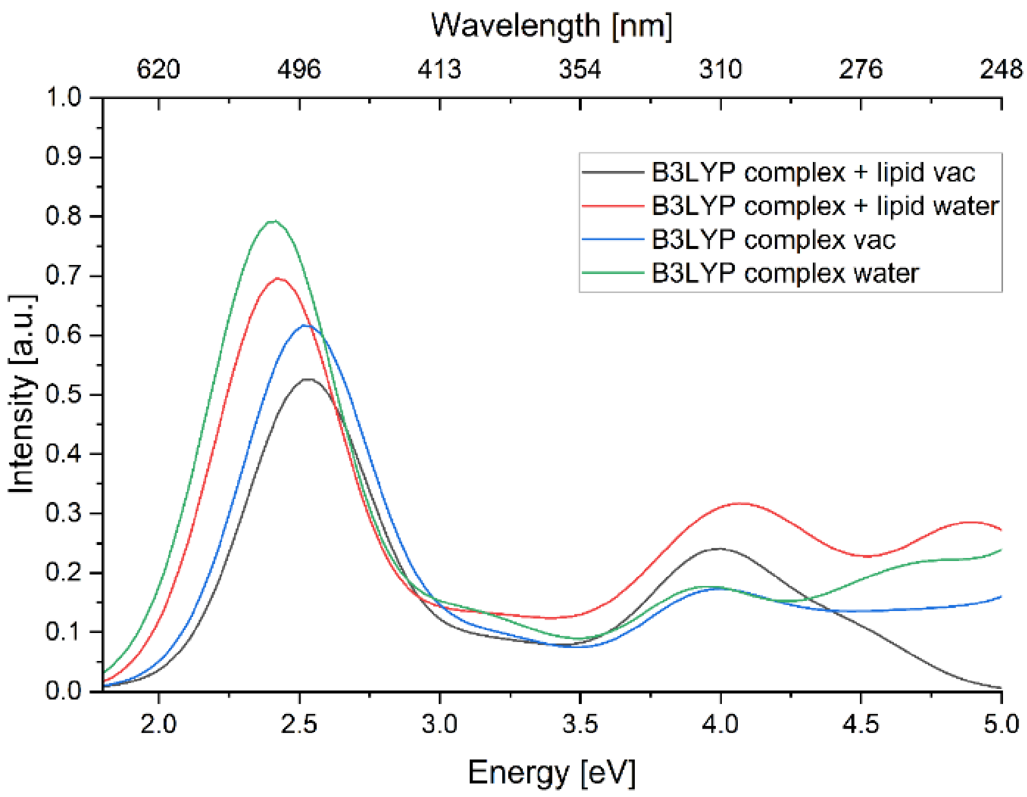

Figure S5. TD-DFT absorption spectra of complex **1** with and without lipid present obtained with the B3LYP functional. It is clearly visible that absorption wavelengths are not influenced by presence of the lipid, just by the chosen environment. The lipid's excited states do not mix with those of the transition metal complex; they sit at much higher energies and are mostly dark. Therefore, omission of the lipid molecule in the TD-DFT calculations was deemed reasonable to lower the computational cost. Extensive benchmarking of the absorption of **1** was carried out.

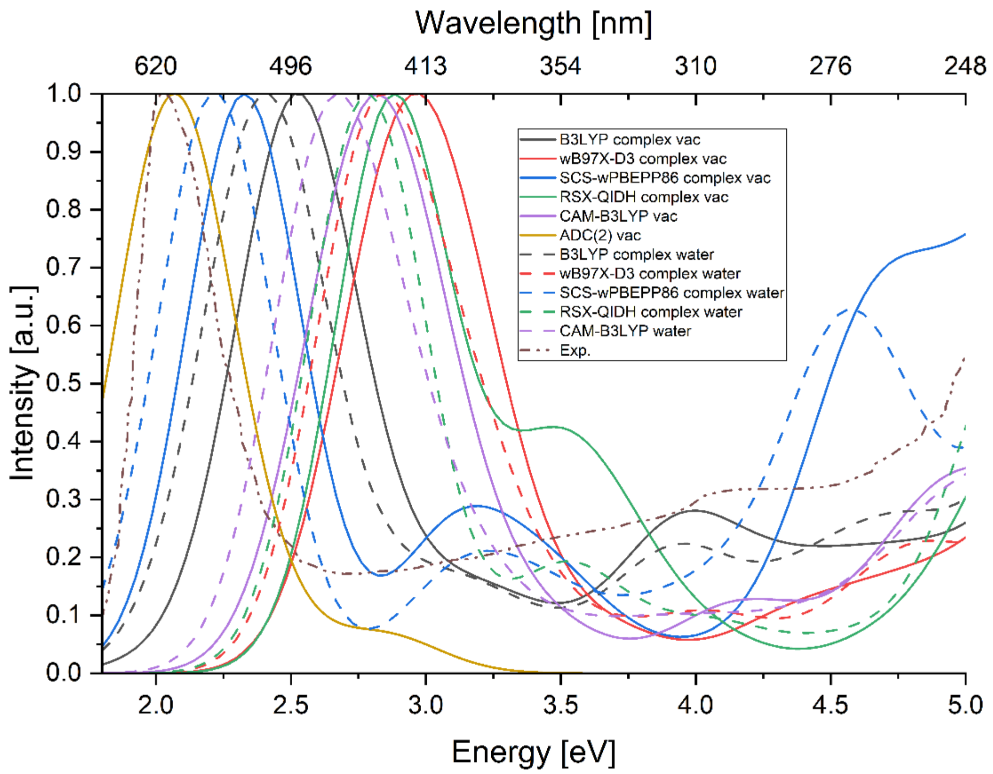

Figure S6. Absorption spectra of **1** in vacuum and water for various DFT functionals and ADC(2).The best fit for absorption is the wavefunction-based method ADC(2) in vacuum. However, the latter could not be used to model luminescence, therefore the range-separated hybrid functional ωB97X-D was chosen.

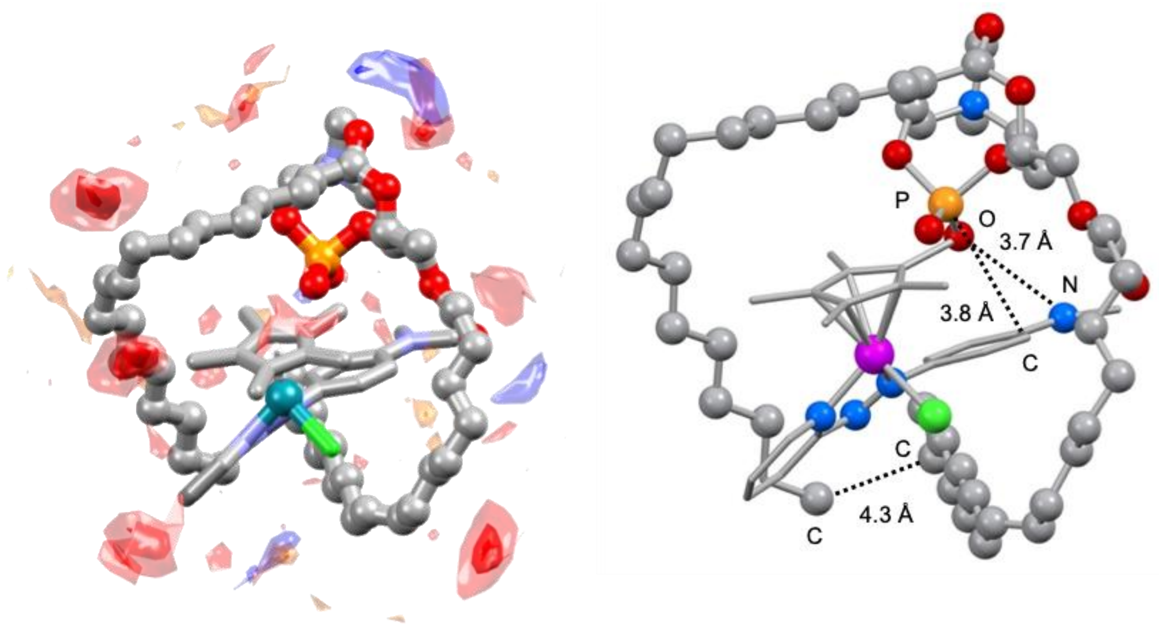

Figure S7. DFT model of DOPC wrapped around **1**. *Left* interaction map. Red, potential H-bond acceptors; brown, hydrophobic regions; blue, H-bond donor areas zones near lipid phosphate and ester groups. *Right* optimised DFT model for correlation, showing selected bond lengths. H atoms omitted for clarity. Deposited file: DOPC_hydrophobic_interaction.pdb.

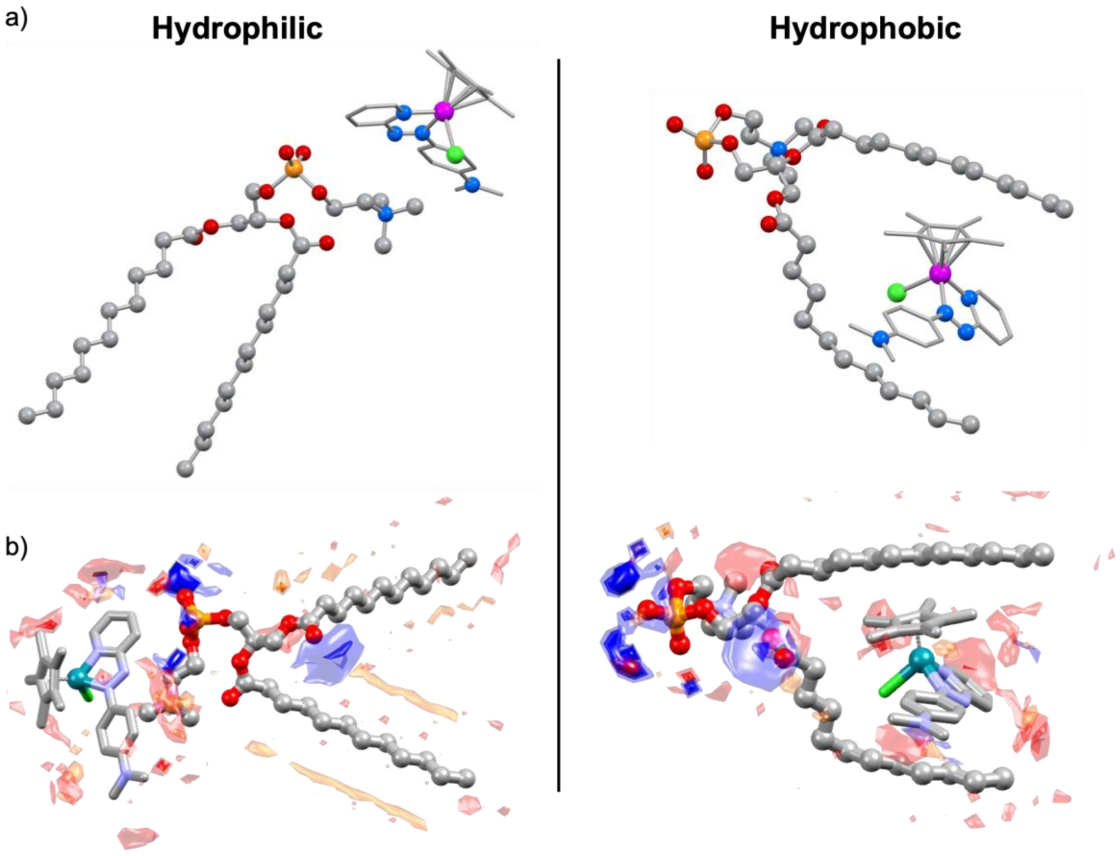

Figure S8. Optimised DFT showing a) hydrophilic and b) hydrophobic interaction of the lipid 1,2-dilauroyl-sn-glycero-3-phosphocholine (DLPC) and **1**. Interaction maps show: red, potential H-bond acceptors; brown, hydrophobic regions; blue, H-bond donor areas zones near lipid phosphate and ester groups. Deposited files: DLPC_hydrophilic_interaction.pdb; DLPC_hydrophobic_interaction.pdb.

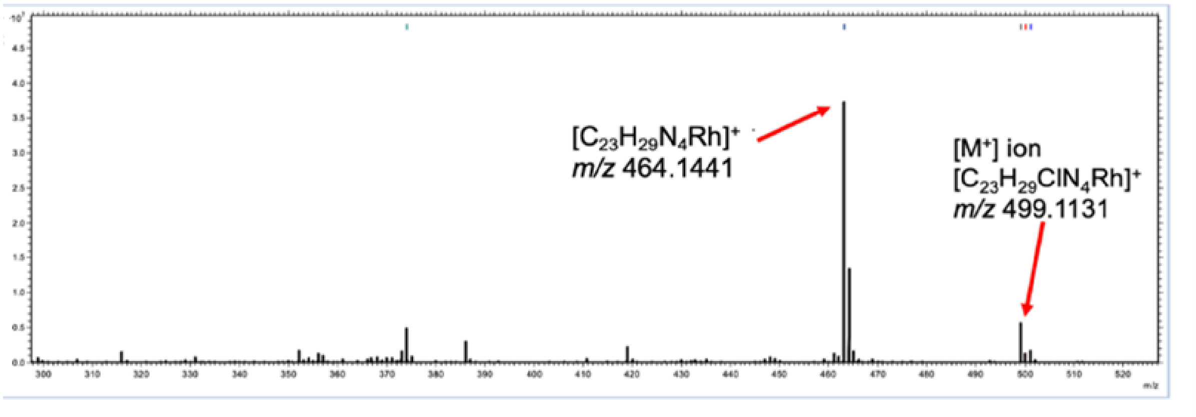

Figure S9. Representative OrbiSIMS spectrum showing ions detected for a control silicon grid treated with complex **1** (in 20% MeOH/80% H_2O, v/v) with peaks at m/z 46.144 assigned to [**1**-HCl]$^+$ and m/z = 499.1131 for [**1**]$^+$.

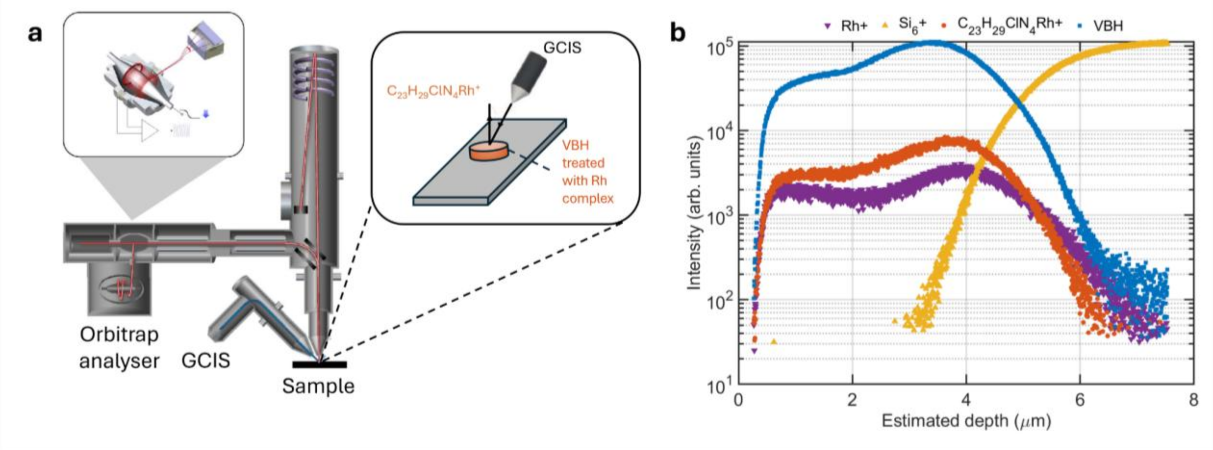

Figure S10. Penetration of complex **1** into a model tissue. a) Schematic of the OrbiSIMS instrument showing the gas cluster ion source (GCIS), the Orbitrap analyser and a veal brain homogenate (VBH) sample mounted on a silicon wafer substrate. b) OrbiSIMS positive-ion depth profiles obtained using a 20 keV Ar2200$^+$ ion beam. Complex **1** orange, [Rh]$^+$ purple, [Si_6]$^+$ yellow, [VBH] blue, where [VBH] is the sum of ion fragments characteristic of choline headgroups of phosphorylcholine lipids: [$C_5H_{15}PNO_4$]$^+$, [$C_5H_{14}NO$]$^+$, [$C_5H_{12}N$]$^+$.

References

(1) Zhang, W.; Bridgewater, H. E.; Banerjee, S.; Soldevila-Barreda, J. J.; Clarkson, G. J.; Shi, H.; Imberti, C.; Sadler, P. J. Ligand-Controlled Reactivity and Cytotoxicity of Cyclometalated Rhodium(III) Complexes. *Eur. J. Inorg. Chem.* **2020**, *2020* (11–12), 1052–1060.

(2) Fu, Y. Organometallic Osmium Arene Anticancer Complexes, PhD thesis University of Warwick, Coventry, UK, **2011**.

(3) Lant, E. C.; Needham, R. J.; Zhang, Z.; Coverdale, J. P. C.; Clarkson, G. J.; Bagley, I.; Dallmann, R.; Sadler, P. J. Cyclopentadienyl Half-Sandwich Rhodium(III) Azopyridine Anticancer Complexes with Activity Tuned by Ligand Substituents. *ChemCatChem* **2025**, *17* (11), e202401863.

(4) Gaussian 16, Revision C.01, Frisch, M. J.; Trucks, G. W.; Schlegel, H. B.; Scuseria, G. E.; Robb, M. A.; Cheeseman, J. R.; Scalmani, G.; Barone, V.; Petersson, G. A.; Nakatsuji, H.; Li, X.; Caricato, M.; Marenich, A. V.; Bloino, J.; Janesko, B. G.; Gomperts, R.; Mennucci, B.; Hratchian, H. P.; Ortiz, J. V.; Izmaylov, A. F.; Sonnenberg, J. L.; Williams-Young, D.; Ding, F.; Lipparini, F.; Egidi, F.; Goings, J.; Peng, B.; Petrone, A.; Henderson, T.; Ranasinghe, D.; Zakrzewski, V. G.; Gao, J.; Rega, N.; Zheng, G.; Liang, W.; Hada, M.; Ehara, M.; Toyota, K.; Fukuda, R.; Hasegawa, J.; Ishida, M.; Nakajima, T.; Honda, Y.; Kitao, O.; Nakai, H.; Vreven, T.; Throssell, K.; Montgomery, J. A., Jr.; Peralta, J. E.; Ogliaro, F.; Bearpark, M. J.; Heyd, J. J.; Brothers, E. N.; Kudin, K. N.; Staroverov, V. N.; Keith, T. A.; Kobayashi, R.; Normand, J.; Raghavachari, K.; Rendell, A. P.; Burant, J. C.; Iyengar, S. S.; Tomasi, J.; Cossi, M.; Millam, J. M.; Klene, M.; Adamo, C.; Cammi, R.; Ochterski, J. W.; Martin, R. L.; Morokuma, K.; Farkas, O.; Foresman, J. B.; Fox, D. J. Gaussian, Inc., Wallingford CT, **2016**.

(5) Becke, A. D. Density-functional Thermochemistry. III. The Role of Exact Exchange. *J. Chem. Phys.* **1993**, *98* (7), 5648–5652.

(6) Stevens, W. J.; Basch, H.; Krauss, M. Compact Effective Potentials and Efficient Shared-exponent Basis Sets for the First-and Second-row Atoms. *J. Chem. Phys.* **1984**, *81* (12), 6026–6033.

(7) Stevens, W. J.; Krauss, M.; Basch, H.; Jasien, P. G. Relativistic Compact Effective Potentials and Efficient, Shared-Exponent Basis Sets for the Third-, Fourth-, and Fifth-Row Atoms. *Can. J. Chem.* **1992**, *70* (2), 612–630.

(8) Cundari, T. R.; Stevens, W. J. Effective Core Potential Methods for the Lanthanides. *J. Chem. Phys.* **1993**, *98* (7), 5555–5565.

(9) Weigend, F. Accurate Coulomb-Fitting Basis Sets for H to Rn. *PhysChemChem* **2006**, *8* (9), 1057–1065.

(10) Oklješa, A. M.; Brenjo, L. M.; Raičević, V. N.; Klisurić, O. R. Characterization and Conformational Analysis of a Novel Hydrazonoyltetrazole: Crystallographic and Theoretical Investigations of an Unexpected Reaction Product. *J. Mol. Struct.* **2025**, *1320*, 139642.

(11) Pracht, P.; Bohle, F.; Grimme, S. Automated Exploration of the Low-Energy Chemical Space with Fast Quantum Chemical Methods. *PhysChemChemPhys* **2020**, *22* (14), 7169–7192.

(12) Bannwarth, C.; Caldeweyher, E.; Ehlert, S.; Hansen, A.; Pracht, P.; Seibert, J.; Spicher, S.; Grimme, S. Extended Tight-binding Quantum Chemistry Methods. *Wiley Interdiscip. Rev. Comput. Mol. Sci.* **2021**, *11* (2), e1493.

(13) Grimme, S. Exploration of Chemical Compound, Conformer, and Reaction Space with Meta-Dynamics Simulations Based on Tight-Binding Quantum Chemical Calculations. *J. Chem. Theory. Comput.* **2019**, *15* (5), 2847–2862.

(14) Neese, F. Software Update: The ORCA Program System-Version 5.0. *Wiley Interdiscip. Rev. Comput. Mol. Sci.* **2022**, *12* (5), e1606.

(15) Neese, F. The ORCA Program System. *Wiley Interdiscip. Rev. Comput. Mol. Sci.* **2012**, *2* (1), 73–78.

(16) Ekström, U.; Visscher, L.; Bast, R.; Thorvaldsen, A. J.; Ruud, K. Arbitrary-Order Density Functional Response Theory from Automatic Differentiation. *J. Chem. Theory Comput.* **2010**, *6* (7), 1971–1980.

(17) Schäfer, A.; Horn, H.; Ahlrichs, R. Fully Optimized Contracted Gaussian Basis Sets for Atoms Li to Kr. *J. Chem. Phys.* **1992**, *97* (4), 2571–2577.

(18) Dolomanov, O. V; Bourhis, L. J.; Gildea, R. J.; Howard, J. A. K.; Puschmann, H. OLEX2: A Complete Structure Solution, Refinement and Analysis Program. *J Appl. Crystallogr.* **2009**, *42* (2), 339–341. https://doi.org/10.1107/S0021889808042726.

(19) Sheldrick, G. M. SHELXT–Integrated Space-Group and Crystal-Structure Determination. *Acta Crystallogr. A Found Adv.* **2015**, *71* (1), 3–8.

(20) Sheldrick, G. M. Crystal Structure Refinement with SHELXL. *Acta Crystallogr. Section C* **2015**, *71* (1), 3–8. https://doi.org/10.1107/S2053229614024218.

(21) Macrae, C. F.; Bruno, I. J.; Chisholm, J. A.; Edgington, P. R.; McCabe, P.; Pidcock, E.; Rodriguez-Monge, L.; Taylor, R.; van de Streek, J.; Wood, P. A. *Mercury CSD 2.0* – New Features for the Visualization and Investigation of Crystal Structures. *J. Appl. Crystallogr.* **2008**, *41* (2), 466–470.

(22) Kounatidis, I.; Stanifer, M. L.; Phillips, M. A.; Paul-Gilloteaux, P.; Heiligenstein, X.; Wang, H.; Okolo, C. A.; Fish, T. M.; Spink, M. C.; Stuart, D. I.; Davis, I.; Boulant, S.; Grimes, J. M.; Dobbie, I. M.; Harkiolaki, M. 3D Correlative Cryo-Structured Illumination Fluorescence and Soft X-Ray Microscopy Elucidates Reovirus Intracellular Release Pathway. *Cell* **2020**, *182* (2), 515-530.e17.

(23) Phillips, M. A.; Harkiolaki, M.; Susano Pinto, D. M.; Parton, R. M.; Palanca, A.; Garcia-Moreno, M.; Kounatidis, I.; Sedat, J. W.; Stuart, D. I.; Castello, A. CryoSIM: Super-Resolution 3D Structured Illumination Cryogenic Fluorescence Microscopy for Correlated Ultrastructural Imaging. *Optica* **2020**, *7* (7), 802–812.

(24) Harkiolaki, M.; Darrow, M. C.; Spink, M. C.; Kosior, E.; Dent, K.; Duke, E. Cryo-Soft X-Ray Tomography: Using Soft X-Rays to Explore the Ultrastructure of Whole Cells. *Emerg. Top. Life Sci.* **2018**, *2* (1), 81–92.

(25) Phillips, M. A.; Harkiolaki, M.; Pinto, D. M. S.; Parton, R. M.; Palanca, A.; Garcia-Moreno, M.; Kounatidis, I.; Sedat, J. W.; Stuart, D. I.; Castello, A. CryoSIM: Super-Resolution 3D Structured Illumination Cryogenic Fluorescence Microscopy for Correlated Ultrastructural Imaging. *Optica* **2020**, *7* (7), 802–812.

(26) Passarelli, M. K.; Pirkl, A.; Moellers, R.; Grinfeld, D.; Kollmer, F.; Havelund, R.; Newman, C. F.; Marshall, P. S.; Arlinghaus, H.; Alexander, M. R. The 3D

OrbiSIMS—Label-Free Metabolic Imaging with Subcellular Lateral Resolution and High Mass-Resolving Power. *Nat. Methods* **2017**, *14* (12), 1175–1183.

(27) Seah, M. P.; Havelund, R.; Gilmore, I. S. Systematic Temperature Effects in the Argon Cluster Ion Sputter Depth Profiling of Organic Materials Using Secondary Ion Mass Spectrometry. *J. Am. Soc. Mass Spectrom.* **2016**, *27* (8), 1411–1418.
